# Production of natural cellulose-based microfibres, from oil palm mesocarp fibres and pineapple leaf wastes, as porous supports for further applications

**DOI:** 10.1016/j.heliyon.2024.e37701

**Published:** 2024-09-10

**Authors:** G.D. Anukwah, V.P.Y. Gadzekpo

**Affiliations:** Department of Chemistry, School of Physical Sciences, College of Agriculture and Natural Sciences, University of Cape Coast, Cape Coast, Ghana

**Keywords:** Purification, Eco-friendly, Cellulosic nature, Gauze bandage, Alkaline-peroxide

## Abstract

Natural cellulose-based microfibers were obtained through an economical and environmentally sustainable process called alkaline-peroxide purification, from the waste products of oil palm mesocarp fibres (OPMF) and pineapple leaves (PL), with the intention of creating porous, biodegradable, biocompatible, and non-toxic solid supports for use in future processes. The extracted microfibres were then taken through microscopic, spectroscopic and thermal characterisation to establish their cellulosic nature. The scanning electron microscopic (SEM) images of the bleached microfibres (B-OPMF and B-PLF) were cleaner, smoother and porous as compared with that of the unrefined fibres (Ur-OPMF and Ur-PLF). The bleached fibres (B-OPMF and B-PLF) exhibited peaks of C and O, which are indicative of pure cellulose, in the energy-dispersive X-ray spectroscopy (EDS) analysis. The FTIR spectral analysis of the extracted cellulose-based fibres (B-OPMF and B-PLF) exhibited peaks that were similar in composition to the reference cellulose (P-GB). For the thermogravimetric analysis (TGA) analysis, the maximum weight degradation in the reference cellulose (P-GB), occurred at 363.11 °C, in the bleached palm fibres (B-OPMF) at 334.55 °C and in the bleached pineapple leaf fibres (B-PLF) at 375.68 °C which, corresponds to cellulose decomposition. The differential scanning calorimetry (DSC) test verified the microfibers' thermally induced transitions. Therefore, these cellulose-based microfibres could be applied as functionalised microfibre supports for future applications.

## 1 Introduction

Researchers are working to replace non-renewable materials with waste from industrial and agricultural processes, as a result of the green economy's transformation of industrial strategies. The creation of new materials from agricultural waste, may be rewarding financially [1,2]. Billion tonnes of lignocellulosic biomass wastes are produced annually, which are mostly disposed off by burning or dumped in landfills. Nonetheless, this growing quantity might function as a cheap source of cellulose, with possible applications [3,4]. For every ton of palm oil produced, 220 kg of oil palm mesocarp fibres are generated. These are mostly lignocellulosic biomass with cellulose (35 %), lignin (44 %), and silica on the fibre surface [2,5]. A significant amount of pineapple trash is produced as a result of the annual harvest of about 30 million tons of pineapple fruit for the food and beverage industries [6,7]. Pineapple wastes have high cellulose content and are rich in fibre [8,9]. In light of this, creative techniques are required to recycle them into beneficial goods with added value.

The intriguing potential of converting oil palm mesocarp fibres and pineapple leaves wastes into cellulose-based microfibres and, consequently, their numerous industrial applications as biomedical materials, composite materials, supercapacitors and polymer matrices have, however, received very little research so far, in reference to a critical review of current literature [[10], [11], [12], [13]]. Therefore, in order to close this enormous gap, more study in the same area is required in the hopes that, oil palm mesocarp fibres and pineapple leaves wastes could be converted into value added products for sustainability. Hence, the objectives of this work is to (i) extract natural cellulose-based microfibres from oil palm mesocarp fibres and fresh pineapple leaf wastes, to be used as support materials for further applications in catalysis and as antimicrobial dressings and (ii) to assess whether the quality of the extracted cellulose-based microfibres, is comparable to that of the reference cellulose (commercial gauze bandage) using microscopic, spectroscopic and thermal characterisation tools.

These natural cellulose-based fibres are safe for human use, environmentally friendly and are regarded as a potential source of renewable energy. They are rich in hydroxyl groups, which can be modified to create other beneficial products, and have demonstrated excellent surface qualities, low density and porosity. As added benefits, they also exhibit notable biological qualities like biodegradability, biocompatibility, and non-toxic nature [[14], [15], [16], [17]]. Novelties based on cellulose have recently been developed to tackle environmental problems, such as heavy metal pollution; absorbents for oil spills and bio-based filters for the treatment of municipal and industrial wastewater [[18], [19], [20]].

Alkaline peroxide extraction is an inexpensive method for extracting cellulose from these wastes and an environmentally friendly way to managing them. Alkaline peroxide's capacity to generate radicals and delignify agriculture wastes without resulting in sugar degradation or furan derivatives makes it a popular reagent in the biomass saccharification, bleaching, and pulping industries [21,22]. Radical species and molecular oxygen are created when the peroxide solution is adjusted to an alkaline state using hydrogen ions. Without a special reaction chamber, this treatment can be performed at room temperature, mild concentration, and atmospheric pressure. Because alkaline peroxide breaks down quickly to produce water and oxygen, it is a "green" reagent with little effect on the environment [23,24].

## 2 Experimental

### 2.1 Materials, reagents and chemicals

Oil palm mesocarp fiber (OPMF) wastes, were collected from a local Oil palm Mill, in Cape Coast, Ghana; pineapple leaves (PL) wastes, from the agriculture farm, university of Cape Coast, Ghana. Absolute Ethanol (100 %), Hydrogen peroxide (30%v/v) and Sodium hydroxide (99 %) were purchased from VWR (BHD Prolabo®) Chemicals, Belgium and used as received.

### 2.2 Preparation and extraction of cellulose from oil palm mesocarp fibres and fresh pineapple leaves

**Fig. 1:**
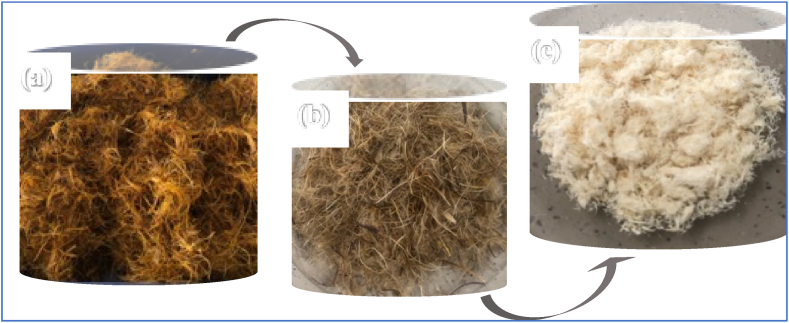
Digital images of the (a) unrefined; (b) hot water treated and (c) bleached oil palm mesocarp fibres.

Cellulose from the fibres was extracted according to a modified method [25]. The unrefined oil palm mesocarp fibres (Figure 1a) were obtained after palm oil was extracted from the pulp at a local oil palm manufacturing company. The fibres were sent to the laboratory, washed with tap water and boiled in tap water at 100 °C for 3 h to remove excessive wax and other impurities. Then, dried in the oven at 85 °C to a constant weight. The dried unrefined fibres (Figure 1b) were then extracted in absolute ethanol at 70 °C for 1.5 h, to further remove oils, waxes and impurities that were insoluble in the hot water but soluble in the ethanol. This process was repeated twice. The fibre-to-solvent ratio was 1:30 (w/v). The dewaxed fibres were then dried in the oven at 85 °C to a constant weight. The dried dewaxed fibres were then boiled in 1 M NaOH solution at 90 °C for 30 min with periodic stirring, to remove lignin and hemicellulose for refined cellulose. This process was repeated two more times with a fibre-to-solvent ratio of 1:30 (w/v). The refined cellulose fibres were then filtered from the NaOH solution and washed with deionised water till neutral pH (pH 7). Then, bleached by immersion in hot 5 % (v/v) H_2_O_2_ at pH 11.5 (pH 11.5, was achieved by the addition of 1 M NaOH) at 90 °C for 45 min, to remove the remaining lignin and whiten the fibres. The process was repeated five more times due to the fibrous nature of the palm fibres, with periodic stirring. The fibre-to-solvent ratio was 1:40 (w/v). The bleached cellulose oil palm mesocarp fibres (B-OPMF) were then filtered from the H_2_O_2_ solution, washed with deionised water till neutral pH (pH 7) and dried in the oven at 85 °C to a constant weight (Figure 1c). Then, stored in labelled air-tight containers for further analysis.

**Fig. 2:**
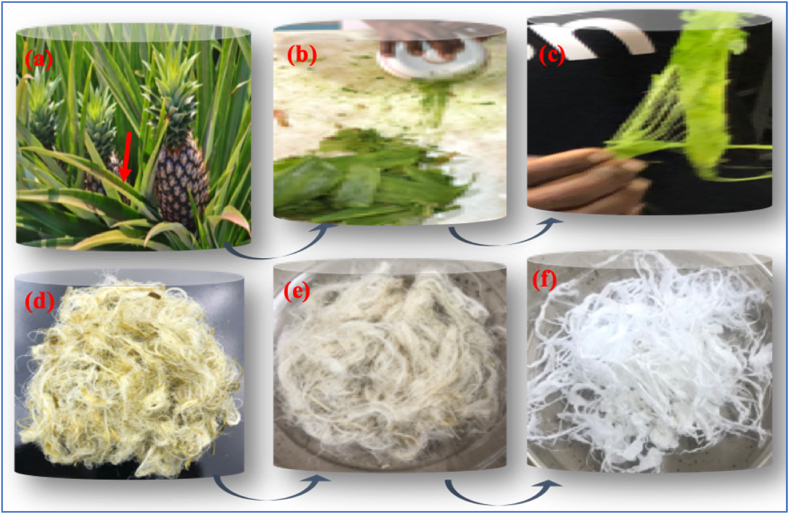
Digital images of (a–c) unrefined pineapple leaves and extracted (d) unrefined; (e) hot water treated and (f) bleached pineapple leaf fibres.

The extraction process of the unrefined pineapple leaves (Ur-PL) differs slightly from that of the unrefined oil palm mesocarp fibres (Ur-OPMF). The pineapple leaf fibres were manually extracted from fresh green pineapple leaves (Figure 2a, red arrow), by using the edge of a ceramic plate to scrape off the green extractable waxy substance from the surface of the leaves (Figure 2b), to expose the long tiny threadlike strips (Figure 2c and d). This process removed most of the lignin content in the fibres exposing more of the α-cellulose. The fibres were then washed severally with tap water at ambient temperature, boiled in tap water at 100 °C for 3 h and then dried in the oven at 85 °C to a constant weight (Figure 2e). The dried fibres were then taken through the same process as that of the oil palm mesocarp fibres to get the bleached cellulose pineapple leaf fibres (B-PLF) in figure 2f.

### 2.3 Preparation of gauze bandage

The reference plain gauze bandage (P-GB) was only boiled in deionised water at 100 °C for 3 h (since, it is an already refined/bleached material). It was dried in the oven at 85 °C to a constant weight and then stored in labelled air-tight containers for further analysis.

## 3 Characterisation of the cellulose-based microfibres

### 3.1 Scanning Electron Microscope-energy-dispersive X-ray spectroscopy (SEM-EDS)

Phenom ProX desktop Scanning Electron Microscope. Manufactured by Thermo Scientific, Eindhoven-the Netherlands was used to view the images of the microfibres. The samples were cut and trimmed to fit the specimen stage and then mounted unto an aluminium stub with a pelco double sided carbon adhesive. An ultra-thin coating of gold was sputtered on the cellulose-based fibres due to poor or no conductivity. Backscattered images were captured at different magnifications (minimum to maximum) using an image intensity and high-resolution voltage mode of 10 kV and a backscatter detector until best image focusing ends.

Also, using the Phenom ProSuite software (element identification), EDS point analysis at 15 kV, duration of 30 s and map analysis at 15 kV, duration of 4 min 26 s were used for the elemental identification, distribution and concentration respectively.

### 3.2 Fourier-transform infrared (FT-IR) spectroscopy

Alpha Platinum ATR FTIR spectrophotometer, manufactured by the Bruker Corporation, Germany, was used to identify the type of functional groups present in the fibres. The unrefined dried fibres and the bleached/refined cellulose-based fibres were placed directly on the crystal plate to coat the entire surface and then a little pressure was applied to the samples to ensure maximum contact. Then, each sample was scanned 24 times to generate a simple spectrum using a software called OPUS.

### 3.3 Thermal Gravimetric Analyzer-differential scanning calorimetry (TGA-DSC)

Simultaneous Thermal Gravimetric Analyzer - Differential scanning calorimetry SDT Q600 V20.9 Build 20. Manufactured by TA Instruments, New Castle, DE, United States of America was used to determine the thermal properties of the microfibres. Approximately 1–2 mg (depending on sample type) of the bleached cellulose-based fibres were put in a crucible and placed in the instrument's sample holder for analysis under nitrogen gas conditions. Measurements were done at 20 °C/min to 800 °C. Data was then generated with the TA universal analysis software.

## 4 Results and discussions

### 4.1 Extraction of cellulose from unrefined fibres

**Fig. 3:**
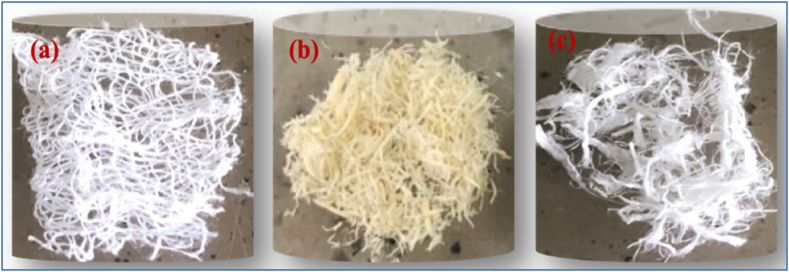
Images of the (a) plain gauze bandage (P-GB); (b) bleached oil palm mesocarp fibres (B-OPMF) and (c) bleached pineapple leaf fibres (B-PLF).

Cellulose-based microfibres were extracted from oil palm mesocarp and pineapple leaf wastes by treatment with alkaline hydrogen peroxide. Hydrogen peroxide (H_2_O_2_) bleaching treatment had a huge effect on fibre brightness, as shown in Fig. 3b and c below. The H_2_O_2_, an oxidising bleach discoloured the fibres. Theoretically, perhydroxyl ions (HOO-) are formed by the dissociation of hydrogen peroxide in alkaline media and are responsible for fibre discolouration. These ions attack the light-absorbing chromophore groups (carbonyl groups, conjugated carbonyl groups, quinones) of lignin and cellulose [26].

The extracted/bleached cellulose-based microfibres from the oil palm mesocarps (B-OPMF) and the pineapple leaves (B-PLF) were whiter in appearance (Fig. 3b & c) as compared to the unrefined (Ur-OPMF and Ur-PLF) fibres (Fig. 1, Fig. 2a) above. This implies that, they have been successfully purified. However, the B-PLF (Figure 3c) appeared to be whiter and softer in nature, as the commercial reference P-GB (Figure 3a) whereas, the B-OPMF was more fibrous in nature and harder (Figure 3b).

### 4.2 Scanning electron microscopic analysis of the unrefined and bleached cellulose-based fibres

**Fig. 4:**
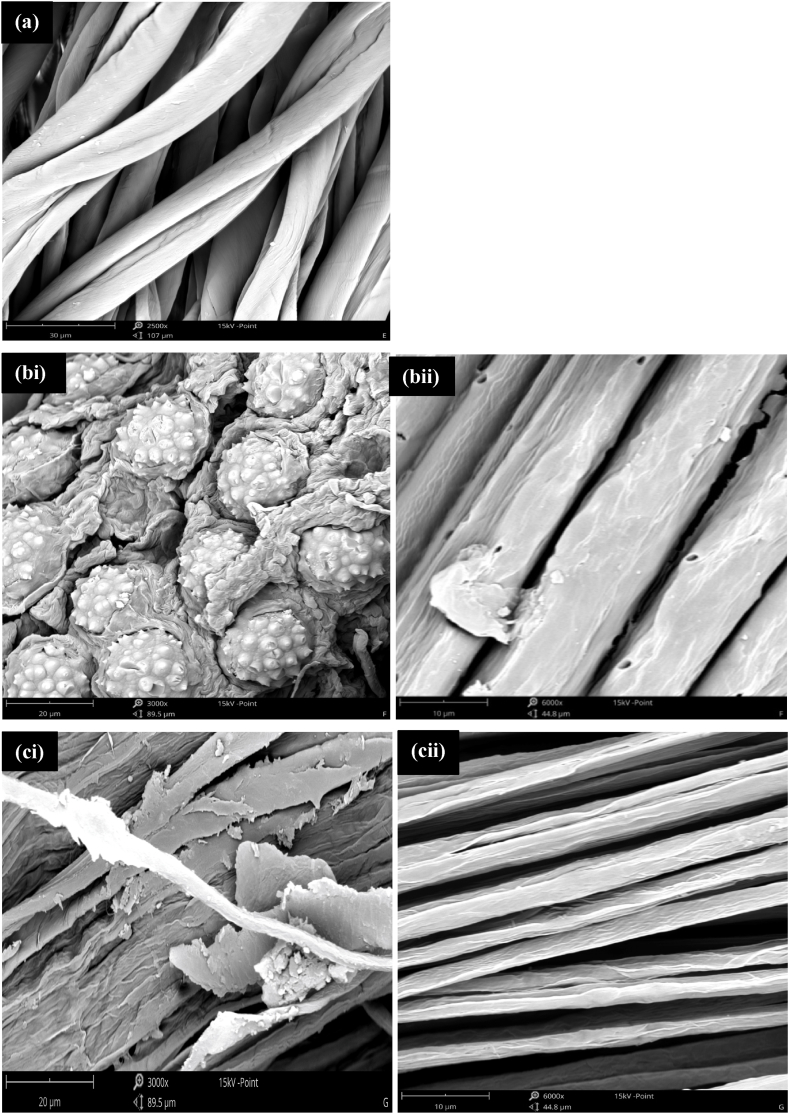
SEM images of the reference fibre (a) P-GB; the unrefined fibres (bi) Ur-OPMF & (ci) Ur-PLF and the Bleached microfibres (bii) B-OPMF & (cii) B-PLF.

The fibre morphologies of the unrefined and the bleached cellulose-based fibres are presented in Figure 4.

In Figure 4, the reference gauze bandage (P-GB) had a morphology, consisting of long cylindrical individual strands, separated from each other (Figure 4a). The external surfaces of the unrefined fibres (Ur-OPMF and Ur-PLF) are rough and covered by layers of non-cellulosic substances and impurities (Fig. 4bi and 4ci). The surface of the Ur-OPMF (Figure 4bi) contain spike-like substances which may be silica particles. Similar observations were also reported in literature [5,27]. After alkaline-peroxide treatment, significant differences in fibre surface morphologies were observed. The surfaces of the bleached/refined microfibres (4 bii and 4 cii) appeared cleaner, smoother, and porous compared to that of the unrefined fibres (4bi and 4ci). The alkaline-peroxide treatment broke the lignocellulosic complex, solubilizing lignin and hemicellulose, exposing more porosity and surface area of the concealed cellulose. Similar results have also been reported by other researchers [17,26,28]. The B-PLF (Figure 4cii) and the reference gauze bandage (P-GB) (Figure 4a) had similar morphology and consist of mostly long cylindrical individual strands, whilst the B-OPMF (Figure 4bii) had short and stacked strands. The surface of the B-OPMF also consists of pores of similar shapes and sizes and some patches. The patches may be residues from some lignin and hemicelluloses which, may not be completely removed during the bleaching and purification processes, due to the fibrous nature of the oil palm mesocarp fibres (OPMF). This was corroborated by the FTIR (Figure 6) and TGA (Figure 7) results. The diameters of the individual strands (micro-sized) within the cellulose-based fibres when measured (not shown) ranged between 9.45 and 16.8 μm for P-GB; 3.31–11.5 μm for B-OPMF and 2.75–5.5 μm for B-PLF.

### 4.3 Energy-dispersive X-ray spectroscopy (EDS) analysis of the cellulose-based microfibres

Energy-dispersive X-ray Spectroscopy (EDS) analysis was conducted to verify the elemental composition of the cellulose-based fibres.

**Fig. 5:**
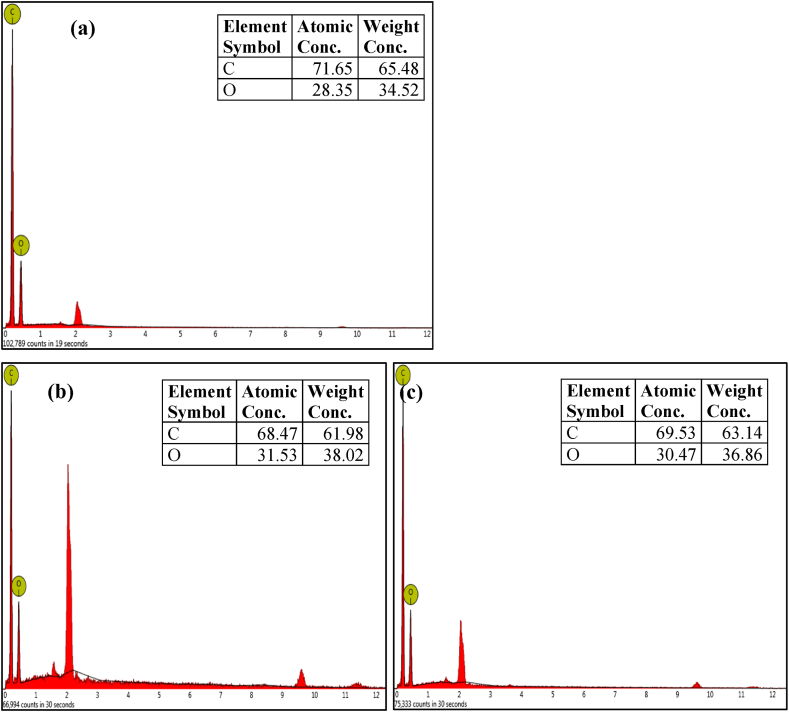
EDS spectra of (a) B-GB, (b) B-OPMF and (c) B-PLF.

From the EDS results in Figure 5, the reference commercial gauze bandage (P-GB) contained mainly of C and O (Figure 5a), which are the characteristic peaks of cellulose. The B-OPMF (Figure 5b) and B-PLF (Figure 5c) also exhibited the same characteristic peaks of C and O. This confirmed the success of the bleaching process and the cellulosic nature of the B-OPMF and B-PLF microfibres as compared with the reference cellulose, P-GB (Figure 5a).

**Fig. 6:**
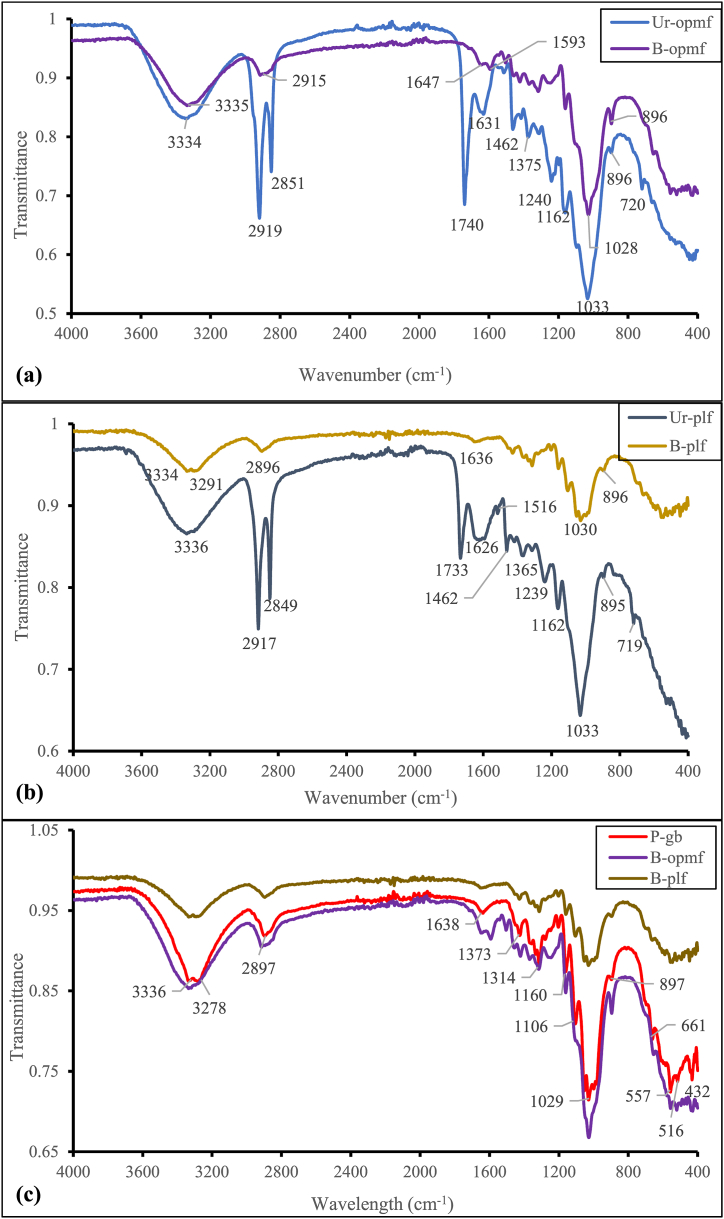
Infra-red spectra of the (a) Unrefined & Bleached oil palm mesocarp fibres (Ur-OPMF & B-OPMF); (b) Unrefined & Bleached pineapple leaf fibres (Ur-PLF & B-PLF) and (c) Bleached/Plain cellulose-based microfibres (P-GB, B-OPMF & B-PLF).

**Fig. 7:**
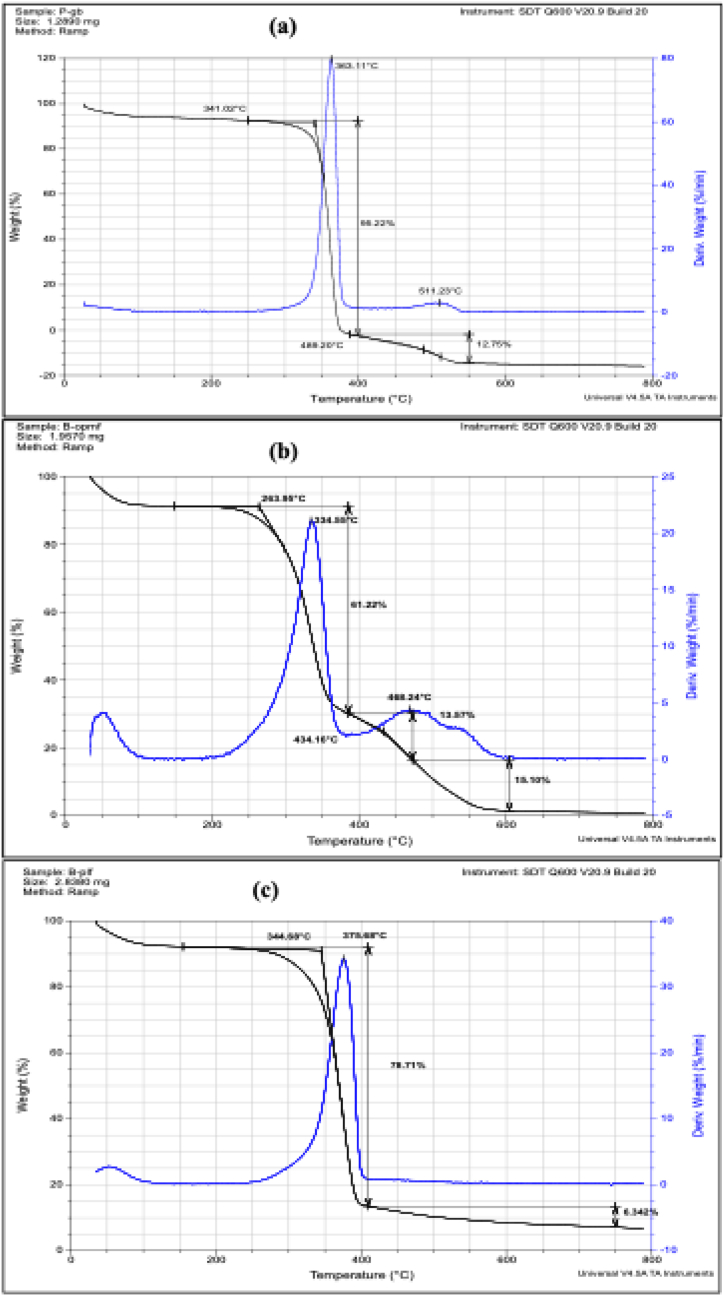
Thermal decomposition curves of the plain cellulose-based fibres (a) P-GB; (b) B-OPMF and (c) B-PLF showing weight losses.

### 4.4 Infra-red spectral analysis of the unrefined and bleached cellulose-based fibres

The infra-red spectra of the unrefined and bleached/refined cellulose-based fibres were determined, to identify the type of functional groups present in them. Also, to confirm whether pure cellulose was successfully extracted from the unrefined oil palm mesocarp fibres and the fresh pineapple leaves. Figure 6 presents the results.

Figure 6 shows the FT-IR spectra of unrefined (Ur-PLF & B-PLF), bleached (B-OPMF & B-PLF*)*, and reference microfibres (P-GB). Bleaching the unrefined fibres has been reported to produce high-quality cellulose fibres [29]. The broad peaks between 3278 and 3336 cm^−1^ in all the fibres (Figure 6a–c) are attributed to the presence of hydroxyl groups (O-H). The two peaks in the unrefined Ur-OPMF (figure 6a) at 2919 cm^−1^ and 2851 cm^−1^, attributed to C–H stretching, were reduced to one peak in the refined B-OPMF at 2915 cm^−1^, and those in the unrefined Ur-PLF (figure 6b) at 2917 cm^−1^ and 2849 cm^−1^ to one at 2896 cm^−1^ in B-PLF. The refined microfibers (P-GB, B-OPMF & B-PLF) [Figure 6c, Table 1] lacked the extended peaks in Ur-OPMF at 1740 cm^−1^ and 1631 cm^−1^ and those in Ur-PLF at 1733 cm^−1^ and 1626 cm^−1^ (Fig. 6a & b), which were attributed to the C=O stretching of the acetyl and ester groups in hemicellulose and aromatic lignin components [30,31]. This demonstrates that the majority, if not all, of the lignin and hemicellulose from the unrefined fibres have been removed, leaving only pure cellulose microfibers. The decrease in the intensity of the peaks around 1240 cm^−1^ in the Ur-OPMF (Fig. 6a) and 1239 cm^−1^ in the Ur-PLF (Figure 6b) is another indication that lignin and hemicellulose were removed during the bleaching process. This is explained by the C–O stretching of the aryl group in lignin and/or the –COO vibration of the acetyl groups in hemicellulose [25]. The peaks around 1626-1647 cm^−1^ in all the fibres (Figure 6a–c) are attributed to the O–H bending of the absorbed water, present in the cellulose, hemicellulose and lignin structures [32]. The peaks at 895-897 cm^−1^ in all the fibres are attributed to the β-glycosidic linkage of cellulose and those at 1028-1033 cm^−1^ to the C-O groups of the cellulose. Compared to the B-OPMF, the IR of the B-PLF resembled the commercial reference P-GB more. This could be explained by the fibrous nature of the Ur-OPMF as a result, the bleaching process might not completely remove the hemicellulose and lignin. The TGA (Figure 7) analysis, which showed the purity in terms of their thermal stability, validated and confirmed this. Overall, the FTIR spectra demonstrated that, the refined cellulose-based microfibres (B-OPMF and B-PLF) were effectively separated from the unrefined fibres (Ur-OPMF and Ur-PLF), and that their functional group compositions were comparable to those of the reference gauze bandage (P-GB)**.**

**Table 1:** Transmittance Peaks (cm^−1^) of the Cellulose-Based Microfibres and their Functional Group Divisions [33].

Ur-OPMF	B-OPMF	Ur-PLF	B-PLF	P-GB	Division
3334	3335	3336	3291/3332	3278/3336	O-H Stretching
2919/2851	2915	2917/2849	2896	2897	C-H Stretching
1740	–	1733	–	–	C=O Bond vibrations of lignin
1631	1647	1626	1636	1638	O-H bending of absorbed water
1512	1593	1516	–	–	C=C Plane symmetric stretching of lignin
1248	–	1239	–	–	C-O-C pyranose ring stretching
1033	1028	1033	1030	1029	C-O group of Cellulose
896	896	895	896	897	C-H out of plane deformation

*** Ur-OPMF: unrefined oil palm mesocarp fibres; B-OPMF: bleached oil palm mesocarp fibres; Ur-PLF: pineapple leaf fibres; B-PLF: bleached pineapple leaf fibres and P-GB: plain gauze bandage (reference cellulose).

Table 1 presents, transmittance peaks of the cellulose-based fibres and their functional group divisions.

### 4.5 Thermogravimetric analyses (TGA) of the cellulose-based microfibres

The TGA curves of cellulose-based microfibres (P-GB, B-OPMF and B-PLF) were measured to determine the thermal events that occurred. The temperature was between 0 °C. and 800 °C., the heating rate in nitrogen atmosphere was 20 °C./min, and the purge rate was 100.0 ml/min.

As observed in Figure 7, the reference and the extracted cellulose-based microfibres (P-GB, B-OPMF and B-PLF) showed component degradation when heated. The initial weight loss seen in all the microfibres between 50 and 100 °C**,** may be the result of the samples drying out from the evaporation of other volatile compounds and absorbed water [32]. The presence of the absorbed water was confirmed by the FT-IR results. P-GB and B-OPMF (Fig. 7a & b) displayed two distinct thermal events, around 350–400 °C and 400–600 °C for P-GB and 350–400 °C and 400–600 °C for B-OPMF whereas, B-PLF (Figure 7c) displayed only one at around 300–400 °C. All three microfibres experienced significant weight loss in the 250–400C region because, the hemicellulose and glycosidic bonds of the cellulose were broken into smaller units [34].

The two prominent peaks displayed by B-OPMF (Figure 7b), one located at 334.55 °C, is associated with the dehydration, decarboxylation, depolymerization, and degradation of cellulose's glycosyl units [32]; and the second, located at 468.24 °C, which is indicative of the degradation of lignin, can occur simultaneously with other degradation steps because of its intricate structure [25]. Due to B-OPMF's fibrous nature, hemicellulose and lignin [35] could not be entirely eliminated during the extraction and bleaching processes, which may account for its' lower starting temperature of 263.95 °C. These outcomes concur with those obtained from the FT-IR. The only peak displayed by the bleached B-PLF at 375.68 °C (Figure 7c), is indicative of the dehydration, decarboxylation, and depolymerization processes as well as the breakdown of cellulose glycosyl units [32]. This implies that the B-PLF that was extracted is extremely pure; if there were impurities, different phases would have been generated. The B-PLF did not exhibit the degradation stages associated with lignin (>390 °C) and hemicelluloses (>180 °C) [31]. The high weight loss with linearity of the thermal curve from 345 to 376 °C and the commercial P-GB (341–363 °C) are comparable. Furthermore, the thermal stability of B-PLF (376 °C) was superior to that of commercial P-GB (363 °C) and B-OPMF (335 °C). This could be due to the fact that B-PLF's cellulose has a higher degree of crystallinity [32] than the reference P-GB since, hemicellulose and lignin components are completely removed during the extraction process, yielding better-quality cellulose. Thus, these conclusions are consistent with the FTIR data.

The maximum weight degradation in the reference cellulose (P-GB), occurred at 363.11 °C, with a 95.22 % weight loss from 350 to 400 °C; in the bleached palm fibres (B-OPMF) at 334.55 °C, with a 61.22 % weight loss from 250 to 400 °C and in the bleached pineapple leaf fibres (B-PLF) at 375.68 °C with a 78.71 % weight loss from 250 to 400 °C (Figure 7a–c) which, corresponds to cellulose decomposition. This is consistent with literature [24,36]. Thus, at temperatures above 300 °C, the cellulose-based microfibres showed notable weight losses, demonstrating their high thermal stability, a crucial prerequisite for many photocatalytic applications.

### 4.6 Differential scanning calorimetry analysis of the cellulose-based microfibres

**Fig. 8:**
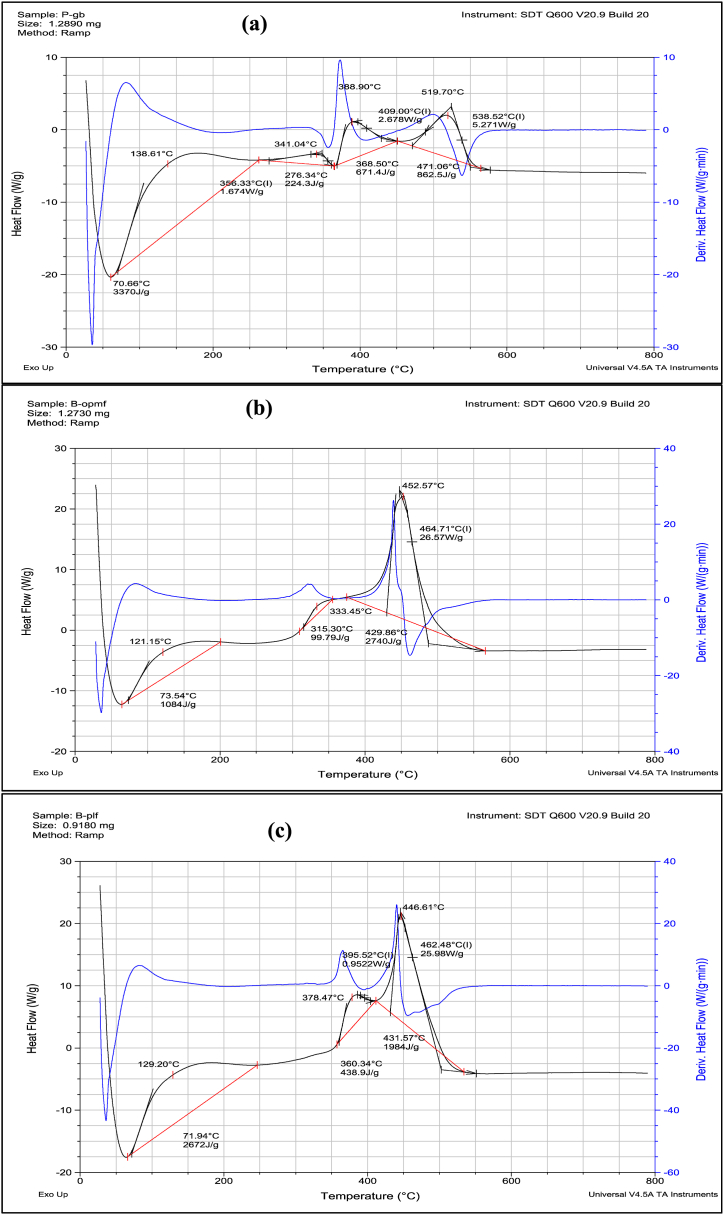
Glass transition temperatures, (Tg) and decomposition enthalpies (Cp) of (a) P-GB (b) B-OPMF and (c) B-PLF.

Temperature changes in exothermic, endothermic, and heat capacity reactions can be measured quantitatively and qualitatively using the differential scanning calorimetry (DSC) technique [37]. In order to ascertain the melting points (Tm), glass transition temperatures (Tg), and enthalpies (Cp) of the thermal events, the DSC curves of the cellulose-based microfibres (P-GB, B-OPMF, and B-PLF) were measured. Figure 8 presents the findings.

As observed in Figure 8, the reference and the extracted cellulose-based microfibres (P-GB, B-OPMF and B-PLF) were stable below 50 °C [32]. The curves (Figure 8a–c) suggest that there aren't any melting points; instead, they appear to break down upon heating, in line with the TGA analysis [34]. When the fibres were heated, there were different areas where they released more heat (exothermic) [38]. The glass transition temperatures (Tg) were shown by the reference cellulose (P-GB) at 356.33 °C, 409.00 °C, and 538.52 °C (Figure 8a), by B-OPMF at 464.71 °C (Figure 8b), and by B-PLF at 395.52 °C and 462.48 °C (Figure 8c). The P-GB displayed four different decomposition products/stages, with total energy of 5128.2 J/g whilst, B-OPMF and B-PLF have three, with total energy of 3920.8 J/g and 5.094.9 J/g respectively (Figure 8a–c, Table 2). Table 2 shows, the glass transition temperatures, Tg, of P-GB, B-OPMF and B-PLF when heated, the temperatures at which they decompose into different components, the maximum decomposition temperature and the heat absorbed during the thermal processes.

**Table 2:** Glass Transition Temperatures (Tg), the Minimum and Maximum Temperatures (T_1_ and T_2_) of Decomposition, the Heat Flow (ΔH) and the Heat absorbed (C_p_) during the Thermal Event of the Cellulose-Based Fibres.

Sample	Tg (^o^C)	ΔH (W/g)	T_1_ (^o^C)	T_2_ (^o^C)	C_p_ (J/g)
**P-GB**			70.66	138.61	3370
	356.33	1.674	276.34	341.54	224.3
	409.00	2.678	368.50	388.90	671.4
	538.52	5.271	471.06	519.70	862.5
**B-OPMF**			73.54	121.15	1084
			315.30	333.45	99.8
	464.71	26.57	429.86	452.57	2740
**B-PLF**			71.94	129.20	2672
	395.52	0.9522	360.34	378.47	438.9
	462.48	25.98	431.57	446.61	1984

*** P-GB: plain gauze bandage (reference cellulose); B-OPMF: bleached oil palm mesocarp fibres; and B-PLF: bleached pineapple leaf fibres.

## 5 Conclusion

Overall, the results from the microscopic, spectroscopic and thermal characterisation confirmed that, pure cellulose was successfully extracted from the raw fibre wastes, and the quality is comparable to that of the reference cellulose (i.e. commercial gauze bandage).

### 5.1 Limitations, future scope and applications

The research evaluation and conclusions are laboratory-based experimental data and their respective analyses.

These natural cellulose-base fibres are porous in nature and have large surface area therefore, could be used as functionalised microfibre supports for photocatalysis and antibacterial wound dressings.

## Data availability

The datasets used and analysed during this study are available from the corresponding author upon reasonable request.

## CRediT authorship contribution statement

**G.D. Anukwah:** Writing – review & editing, Writing – original draft, Resources, Methodology, Investigation, Funding acquisition, Formal analysis, Conceptualization. **V.P.Y. Gadzekpo:** Writing – review & editing, Writing – original draft, Supervision, Conceptualization.

## Declaration of competing interest

The authors declare that they have no known competing financial interests or personal relationships that could have appeared to influence the work reported in this paper.
